# Effects of caffeine supplementation on physical performance and mood dimensions in elite and trained-recreational athletes

**DOI:** 10.1186/s12970-019-0332-5

**Published:** 2020-01-03

**Authors:** P. Jodra, A. Lago-Rodríguez, A. J. Sánchez-Oliver, A. López-Samanes, A. Pérez-López, P. Veiga-Herreros, A. F. San Juan, R. Domínguez

**Affiliations:** 1 Faculty of Health Sciences, Universidad Alfonso X El Sabio, Madrid, Spain; 2 University of Alcalá, Madrid, Spain; 3 Faculty of Health Sciences, Universidad Isabel I, Burgos, Spain; 4 Department of Human Motricity and Sports Performance, Faculty of Education Sciences, Seville University, Seville, Spain; 5 School of Physiotherapy, Faculty of Health Sciences, Universidad Francisco de Vitoria, Madrid, Spain; 6 Department of Biomedical Sciences, Faculty of Medicine and Health Sciences, University of Alcalá, Madrid, Spain; 7 Sports Biomechanics Laboratory, Department of Health and Human Performance, Faculty of Physical Activity and Sport Sciences – INEF, Universidad Politécnica de Madrid, Madrid, Spain

**Keywords:** Caffeine, Sport supplementation, Athletes, Boxing, Ergogenic aid

## Abstract

**Background:**

Caffeine supplementation (CAFF) has an established ergogenic effect on physical performance and the psychological response to exercise. However, few studies have compared the response to CAFF intake among athletes of different competition level. This study compares the acute effects of CAFF on anaerobic performance, mood and perceived effort in elite and moderately-trained recreational athletes.

**Methods:**

Participants for this randomized, controlled, crossover study were 8 elite athletes (in the senior boxing national team) and 10 trained-recreational athletes. Under two experimental conditions, CAFF supplementation (6 mg/kg) or placebo (PLAC), the athletes completed a Wingate test. Subjective exertion during the test was recorded as the rating of perceived exertion (RPE) both at the general level (RPE_general_) and at the levels muscular (RPE_muscular_) and cardiorespiratory (RPE_cardio_). Before the Wingate test, participants completed the questionnaires Profiles of Moods States (POMS) and Subjective Vitality Scale (SVS).

**Results:**

In response to CAFF intake, improvements were noted in W_peak_ (11.22 ± 0.65 vs 10.70 ± 0.84; *p* = 0.003; ${\eta}_p^2$ *=0*.44), W_avg_ (8.75 ± 0.55 vs 8.41 0.46; *p* = 0.001; ${\eta}_p^2$ =0.53) and time taken to reach W_peak_ (7.56 ± 1.58 vs 9.11 ± 1.53; *p* <  0.001; ${\eta}_p^2$ =0.57) both in the elite and trained-recreational athletes. However, only the elite athletes showed significant increases in tension (+ 325%), vigor (+ 31%) and SVS (+ 28%) scores after the intake of CAFF compared to levels recorded under the condition PLAC (*p* <  0.05). Similarly, levels of vigor after consuming CAFF were significantly higher in the elite than the trained-recreational athletes (+ 5.8%).

**Conclusions:**

CAFF supplementation improved anaerobic performance in both the elite and recreational athletes. However, the ergogenic effect of CAFF on several mood dimensions and subjective vitality was greater in the elite athletes.

## Background

Caffeine (CAFF) is a nutritional supplement with a high level of scientific evidence supporting its effect of improving sports performance [1]. This explains why CAFF has become one of the most widely consumed nutritional aids among athletes [2, 3], both professional [4, 5] and recreational/amateur [6, 7]. Moreover, its popularity has also increased since it was removed from the list of banned substances for sports in 2004 [8].

As CAFF is molecularly similar to adenosine, it blocks adenosine receptors A_1_, A_2a_ and A_2b_ [9], acting as a powerful central nervous system stimulator [10], and upregulating the synthesis of catecholamines with neurotransmitter activity (e.g., dopamine, epinephrine and norepinephrine) [11, 12]. As skeletal muscle has many adenosine receptors [13], CAFF intake increases neuromuscular recruitment [14, 15]. Further, at the muscle tissue level, CAFF increases the frequency of calcium channel opening [16], promoting the release of calcium into the myoplasm and thus enhancing muscle contraction [17]. These effects make CAFF an effective ergogenic aid in various exercise actions such as endurance sports [18], exercise efforts with high glycolytic demands [19], resistance exercise [20], and racket [21], combat [22] and team sports [23].

Besides increasing catecholamine levels, the effects of CAFF supplementation on the central nervous system include increased sympathetic activity and reduced parasympathetic activity. In turn, these effects modify a person’s mood [24, 25], enhancing alertness and reducing the feeling of fatigue [26, 27], such that there is a subjective perception of vitality when faced with a physical activity task. Increased perceived tension reflects optimization of the state of preparation of an athlete to undertake a physical test. The relationship between subjective tension and an athlete’s preparation state follows a hump-shaped function, whereby levels of tension that are too low or too high will mean a decline in performance when adjusting to a person’s optimal performance zone [28]. This explains the known effects of CAFF on emotional factors of tension and vigor, increasing their perceived levels and improving an individual’s willingness to tackle the task at hand [29, 30]. In turn, this reduces perceived effort levels (RPE), both in trained and untrained individuals [31].

With the objective of testing whether CAFF consumption is conditioned by an athlete’s training level, Skinner et al. compared blood caffeine concentrations after taking an oral CAFF supplement in untrained subjects versus trained resistance sport athletes [32]. Results indicated that caffeine concentrations were higher in the trained athletes. This suggests that the ergogenic effect of CAFF supplementation could be associated with the training level (trained vs. untrained), and performance level (elite vs. recreational) of the athlete. Evidence supporting this theory is, however, scarce and results have been conflicting [13, 33–37]. Hence, while various studies have examined populations of athletes with different performance levels, so far no study has tried to assess whether an athlete’s training level could determine the ergogenic effects of CAFF supplementation. The present study was therefore designed to compare the acute effects of CAFF supplementation on physical performance and mood when executing anaerobic efforts and to assess the effort perceived by elite athletes and moderately-trained recreational athletes.

## Methods

### Participants

Eighteen men were enrolled. Eight of these participants were elite athletes (age: 22.0 ± 1.8 years; body mass: 65.6 ± 10.8 kg; height: 1.69 ± 0.09 m; BMI: 22.7 ± 1.3 kg/m^2^), members of the Spanish national senior male boxing team who had been training at the High Performance Center in Madrid, Spain, and participating in international competitions for over 2 years. The remaining 10 subjects were undergraduate students from the Department of Sport Sciences of the Universidad de Alfonso X El Sabio, Madrid, Spain (age: 22.5 ± 1.8 years; body mass: 75.0 ± 10.8 kg; height: 1.75 ± 0.04 m; BMI: 24.3 ± 2.6 kg/m^2^). These subjects were categorized as recreational athletes based on the following criteria: (a) at least 3 years of experience with strength training, having completed within the past 18 months ≥3 sessions/week without interruption; (b) a bench press one-repetition maximum (1 RM) greater than body weight, and a full squat 1 RM 1.5 times body weight [38]; (c) no nutritional supplements taken in the 3 months before the study outset; (d) no smoking; (e) no disease or other impediment that could affect cycle ergometry performance.

Participants in both experimental groups were volunteers who signed up for this study after an informative session held 1 week before the study outset. Candidates were first informed of the possible secondary effects of CAFF supplementation. The study protocol fulfilled the tenets of the Declaration of Helsinki, and was approved by the Ethics Committee of the Universidad Alfonso X El Sabio.

### Experimental design

The study design was crossover, randomized, double-blind, placebo-controlled. Each participant undertook two test sessions within 48 h at the university Exercise Physiology laboratory at the same time of day (±0.25 h). In each session, 50% of the athletes were randomly assigned a CAFF (6 mg/kg) or placebo (PLAC) (6 mg/kg of sucrose) supplement.

Upon arrival at the laboratory for each session, participants were given a supplement (CAFF or PLAC) and after a 60 min period of rest, they completed the questionnaires profile of mood states (POMS) and subjective vitality scale (SVS). After a standardized warm-up, the subjects performed a Wingate test on a cycle ergometer to assess anaerobic performance [39]. Immediately after the test, they graded their exertion using the rate of perceived exertion (RPE) scale.

### Nutritional intervention

Caffeine supplements were provided in #1 non-transparent red capsules (Guinama S.L.U, 0044634, La Pobla de Valbona, Spain). Individual capsules were prepared based on each participant’s body weight, so each capsule supplied 6 mg/kg of either CAFF or PLAC to each participant. Capsules were prepared following the standard work procedure described in the *Formulario Nacional Español* using a semi-automatic manual filling machine Capsunorm 2000 (Miranda de Ebro, Spain). The timing of supplement intake was based on the fact that peak blood caffeine levels are reached 1 h post-ingestion [40] and on the results of a disaggregation quality assay described in the *Real Farmacopea Española* (2005) of 13.4 min [41].

In line with previous research [42], subjects were provided with a set of guidelines to ensure each individual ingested similar proportions of carbohydrates (60%), lipids (30%) and proteins (10%), aiming at avoiding interactions between the supplementation and any nutritional factor. The intake of caffeine was also restricted 24 h before the study start and the subjects were provided with a list of foodstuffs rich in caffeine (coffee, tea, mate, energizing drinks, cola drinks, chocolate drinks and chocolate) they should avoid.

### Profile of mood states (POMS)

To assess the participants’ mood, we used the profile of mood states (POMS) questionnaire in its original reduced version [43], translated into Spanish and validated by Fuentes et al. [44]. Participants graded a set of 29 items related to mood on a Likert scale from 0 (not at all) to 4 (extremely) in reply to the question “How do you feel at this moment?” to assess six scales: tension, depression, anger, vigor, fatigue and confusion.

### Subjective vitality scale (SVS)

Participants’ vitality was assessed using the Spanish version of the subjective vitality scale (SVS) [45] of Balaguer et al. [46]. Subjects are required to indicate their agreement with seven statements related to subjective feelings of energy and vitality using a 7-point Likert scale where 1 means “total disagreement” and 7 means “total agreement”.

### Anaerobic performance

A Monark cycle ergometer (Ergomedic 828E, Vansbro, Sweden) was used for the Wingate test. The test was preceded by a standardized warm up as described previously [42, 47] and consisted of 30 s of cycling at maximum effort with a load (Kp) corresponding to 7.5% of the subject’s body weight. The test was started from a stop position and the first round of pedaling initiated with the dominant leg. Participants were encouraged to reach the maximum rpm in the shortest time possible and try to maintain this pedaling speed until the end of the test. Throughout the test, the athletes were motivated by 5 investigators.

Power (W) was recorded during each second of the test. The following variables were subsequently calculated: the highest W value recorded during the test or peak power (Wpeak), the time in seconds (s) taken to reach Wpeak (Time Wpeak), mean W for the test duration (Wmean) and minimum power (Wmin), taken as the lowest W recorded during the last 10 s of the test.

### Rating of perceived exertion (RPE)

In line with previous research [48], a 6 to 20 RPE scale of Borg [49] was presented as soon as the Wingate test was finished. Accordingly, participants were first asked to report RPE regarding muscular pain felt at legs (RPE_muscular_); second, participants were asked to report RPE only at cardiorespiratory level (RPE_cardio_); and finally, participants had to declare global RPE (RPE_general_), which included features from both muscular and cardiorespiratory dimensions.

### Statistical analysis

Data are presented as means ± standard deviations (SD). The normal distribution of data was tested using the Kolmogorov-Smirnov test, and equality of variances was established with the Levene’s test. When an inequality of variances was found, a non-parametric test was used, in which case the value of the adjusted test statistic and degrees of freedom are reported. To ensure similar anthropometric and personal variables between elite and trained-recreational athletes, separate Student t-tests for independent samples (elite vs. trained-recreational) were run for age, weight, height, and body mass index (BMI), respectively.

To compare the effects of CAFF supplementation on physical and psychological measures between the two groups of athletes, separate 2 × 2 independent analyses of variance for repeated measures (ANOVA-RM) were applied for each variable recorded. Performance level (elite vs. trained-recreational) was introduced as an inter-subject factor, whereas Supplementation (CAFF vs. PLAC) was used as an intra-subject factor. Practical significance for pairwise comparisons was assessed by calculating Cohen’s *d* effect size [50]. Effect sizes (d) of above 0.8, between 0.8 and 0.5, between 0.5 and 0.2 and lower than 0.2 were considered as large, moderate, small, and trivial, respectively [51]. Further, ANOVA-RM effect sizes were calculated using partial eta squared (${\eta}_p^2$), and <  0.25, 0.26–0.63 and > 0.63 considered small, medium and large effect sizes respectively [52, 53]. All statistical tests were performed using the Statistical Package for Social Sciences (version 20.0 for Mac, SPSS™ Inc., Chicago, IL, USA). Significance was set at *p* <  0.05.

## Results

No significant differences between experimental groups (elite vs. trained- recreational) were detected in the variables age (t_16_ = 0.593; *p* = 0.561; *d* = 0.28); weight (t_16_ = 1.838; *p* = 0.085; *d* = 0.87); height (t_9.41_ = 1.694; *p* = 0.123; *d* = 0.87); and BMI (t_16_ = 1.594; *p* = 0.130; *d* = 0.76).

### Anaerobic performance

The results found for the anaerobic performance measures are summarized in Table 1. A significant effect of the factors supplementation (F_1,16_ = 12.804; *p* = 0.003; ${\eta}_p^2$ *= 0*.44) and group (F_1,16_ = 8.915; *p* = 0.009; ${\eta}_p^2$ = 0.36) was observed when peak power was analyzed. Thus, participants showed higher W_peak_ values after CAFF supplementation compared to placebo (11.22 ± 0.65 vs 10.7 ± 0.84 W) whereas trained-recreational athletes showed a higher W_peak_ (11.31 ± 0.73) than the elite athletes (10.52 ± 0.62). However, there was no significant interaction between supplementation and group (F_1,16_ = 0.652; *p* = 0.431; ${\eta}_p^2$ = 0.04).

**Table 1:** Performance variables recorded in each experimental group

		Elite	Recreational	*p*-values (ES)		
				Supplementation	Group	Supplementation x Group
W_max_	Caffeine	10.85 ± 0.48	11.52 ± 0.62	0.003** (0.44)	0.009** (0.36)	0.431 (0.04)
	Placebo	10.19 ± 0.59	11.11 ± 0.8			
W_avg_	Caffeine	8.69 ± 0.38	8.8 ± 0.67	0.001**(0.53)	0.378 (0.07)	0.290 (0.05)
	Placebo	8.25 ± 0.37	8.54 ± 0.51			
W_min_	Caffeine	6.49 ± 0.22	6.01 ± 1.1	0.452 (0.04)	0.345 (0.06)	0.422 (0.04)
	Placebo	6.19 ± 0.56	6.02 ± 0.89			
T W_max_	Caffeine	8.00 ± 1.6	7.2 ± 1.55	<0.001*** (0.57)	0.783 (0.05)	0.077 (0.18)
	Placebo	8.88 ± 1.64	9.3 ± 1.49			

Data are provided as the mean ± standard deviation. Adjusted values for the test statistic (F) and degrees of freedom (df) are shown when inequality of variances were found between groups

Abbreviations: ES: effect size; W_max_: maximum power; W_avg_: average power; W_min_: minimum power; T W_max_: time to maximum power

**: *p* < 0.01; ***: *p* < 0.001

For the average power exerted by participants during the Wingate test, we observed a significant effect of supplementation (F_1,16_ = 18.099; *p* = 0.001; ${\eta}_p^2$ = 0.531). Participants showed a greater W_avg_ after CAFF supplementation (8.75 ± 0.55) compared to placebo (8.41 ± 0.46). No significant supplementation by group interaction emerged (F_1,16_ = 1.197; *p* = 0.290; ${\eta}_p^2$ = 0.07); nor did we observe a significant effect of group (F_1,16_ = 0.820; *p* = 0.378; ${\eta}_p^2$ = 0.05).

As for minimum power in the Wingate test, no significant supplementation by group interaction was detected (F_1,16_ = 0.680; *p* = 0.422; ${\eta}_p^2$ = 0.04). Neither were any significant effects observed of the factors supplementation (F_1,16_ = 0.595; *p* = 0.452; ${\eta}_p^2$ = 0.04) or group (F_1,16_ = 0.948; *p* = 0.345; ${\eta}_p^2$ = 0.06).

Finally, when we examined the time it took the participants to achieve maximum power, a significant effect emerged of supplementation (F_1,16_ = 21.138; *p* <  0.001; ${\eta}_p^2$ = 0.57). This meant that the athletes reached W_peak_ earlier after CAFF supplementation (7.56 ± 1.58) compared to placebo (9.11 ± 1.53). There was no significant supplementation by group interaction (F_1,16_ = 3.584; *p* = 0.077; ${\eta}_p^2$ = 0.18) or a significant effect of group (F_1,16_ = 0.079; *p* = 0.783; ${\eta}_p^2$ = 0.005).

### Rating of perceived exertion (RPE)

Table 2 details the ratings of perceived exertion awarded by the participants. A significant effect was detected for the factor group (F_1,16_ = 6.507; *p* = 0.021; ${\eta}_p^2$ = 0.29) in the exertion perceived by the athletes in the legs (RPE_muscular_) whereby the trained-recreational athletes showed higher RPE_muscular_ (18.20 ± 1.06) than the elite athletes (15.75 ± 3.17). There were no significant supplementation by group interactions (F_1,16_ = 0.02; *p* = 0.889; ${\eta}_p^2$ = 0.001), or significant effects of supplementation (F_1,16_ = 0.376; *p* = 0.548; ${\eta}_p^2$ = 0.02).

**Table 2:** Ratings of perceived exertion recorded in each experimental group

		Elite	Recreational	p-values (ES)		
				Supplementation	Group	Supplementation x Group
RPE_legs_	Caffeine	15.63 ± 2.82	18.00 ± 1.15	0.548 (0.02)	0.021* (0.29)	0.889 (0.001)
	Placebo	15.88 ± 3.68	18.40 ± 0.97			
RPE_cardio_	Caffeine	14.50 ± 3.62	17.20 ± 1.75	0.419 (0.04)	0.019* (0.30)	0.785 (0.05)
	Placebo	14.75 ± 2.76	17.70 ± 1.57			
RPE_general_	Caffeine	15.50 ± 3.66	17.70 ± 1.16	0.094 (0.16)	0.079 (0.18)	0.846 (0.002)
	Placebo	16.13 ± 3.48	18.20 ± 0.92			

Data are provided as the mean ± standard deviation. Adjusted values for the test statistic (F) and degrees of freedom (df) are shown when inequality of variances were found between groups

Abbreviations: ES: effect size; RPE_legs_: exertion perceived at the level of the legs; RPE_cardio_: exertion perceived at the cardiorespiratory level; RPE_general_: general perceived exertion

*: *p* < 0.05

A significant effect of the factor group was found in exertion perceived at the cardiorespiratory level (F_1,16_ = 6.829; *p* = 0.019; ${\eta}_p^2$ = 0.3) in that the trained-recreational athletes showed a larger RPE_cardio_ (17.45 ± 1.64) than the elite athletes (14.63 ± 3.12). There was no significant supplementation by group interaction (F_1,16_ = 0.077; *p* = 0.785; ${\eta}_p^2$ = 0.005) nor significant effect of the factor supplementation (F_1,16_ = 0.69; *p* = 0.419; ${\eta}_p^2$ = 0.04).

No significant supplementation by group interaction (F_1,16_ = 0.039; *p* = 0.846; ${\eta}_p^2$ = 0.002) was found for the general rate of perceived exertion (RPE_general_). Moreover, there was no significant effect of supplementation (F_1,16_ = 3.172; *p* = 0.094; ${\eta}_p^2$ = 0.16) or group (F_1,16_ = 3.524; *p* = 0.079; ${\eta}_p^2$ = 0.18).

### Psychological measures

**Fig. 1:**
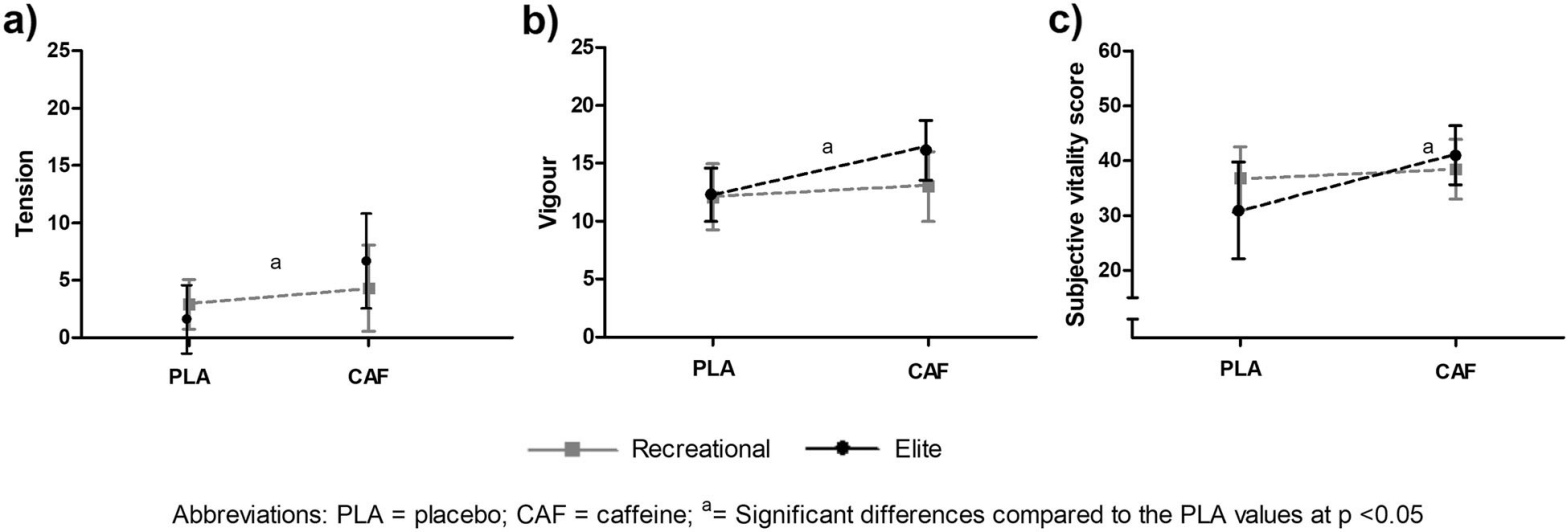
Panel **a** shows the scores recorded for the dimension *tension*. Higher tension scores were reported by elite athletes after supplementation with caffeine compared to placebo; Panel **b** shows the scores recorded for the dimension *vigor*. Scores for vigor were significantly higher after caffeine supplementation in the elite athletes compared to the trained-recreational athletes. Further, elite athletes returned higher vigor scores after supplementation with caffeine compared to placebo; Panel **c** shows the scores recorded in the SVS. Elite athletes showed larger SVS values after supplementation with caffeine versus placebo. *a*: significant differences were detected in elite athletes when caffeine was compared with placebo. *b*: significant differences after caffeine supplementation were detected in elite athletes versus trained-recreational athletes

In Table 3, we provide the results found for the psychological measures. A significant supplementation by group interaction was noted for tension (F_1,16_ = 6.526; *p* = 0.021; ${\eta}_p^2$ = 0.29; Fig. 1a), which was accompanied by a significant effect of the factor supplementation (F_1,16_ = 16.552; *p* = 0.001; ${\eta}_p^2$ = 0.51). However, no significant effect of group was observed (F_1,16_ = 0.815; *p* = 0.380; ${\eta}_p^2$ = 0.05). Our post-hoc pairwise analysis revealed significantly greater tension levels reported by the elite athletes after caffeine intake compared with placebo (8 ± 4.98 vs. 1.88 ± 3.56; *p* <  0.001; *d* = 1.43).

**Table 3:** Psychological measures recorded in each experimental group

		Elite	Recreational	p-values (ES)		
				Supplementation	Group	Supplementation x Group
*Tension*	Caffeine	8.00 ± 4.99^†^	4.30 ± 3.77	0.001** (0.51)	0.380 (0.05)	0.021* (0.29)
	Placebo	1.88 ± 3.56	2.90 ± 2.18			
*Depression*	Caffeine	3.63 ± 0.74	1.00 ± 1.25	0.894 (0.001)	0.036* (0.25)	0.242 (0.008)
	Placebo	2.88 ± 0.99	1.60 ± 3.69			
*Anger*	Caffeine	0.38 ± 1.06	2.40 ± 3.10	0.690 (0.01)	0.180 (0.08)	0.241 (0.11)
	Placebo	0.88 ± 1.81	1.40 ± 2.46			
*Vigor*	Caffeine	16.50 ± 2.62^†#^	13.00 ± 3.02	< 0.001*** (0.64)	0.119 (0.14)	0.004** (0.41)
	Placebo	12.62 ± 2.33	12.10 ± 2.85			
*Fatigue*	Caffeine	2.50 ± 3.50	2.60 ± 2.22	0.008** (0.36)	0.370 (0.05)	0.088 (0.17)
	Placebo	6.50 ± 4.54	3.60 ± 4.09			
*Confusion*	Caffeine	14.63 ± 3.54	12.20 ± 3.99	0.619 (0.02)	0.324 (0.06)	0.236 (0.09)
	Placebo	14.13 ± 4.36	13.40 ± 2.22			
*SVS*	Caffeine	40.63 ± 4.78^†^	38.40 ± 5.44	< 0.001*** (0.59)	0.597 (0.02)	0.004** (0.41)
	Placebo	31.75 ± 7.78	36.80 ± 5.71			

Data are provided as the mean ± standard deviation. Adjusted values for the test statistic (F) and degrees of freedom (df) are shown when inequality of variances were found between groups

Abbreviations: SVS: subjective vitality scale; ES: effect size

*: *p* < 0.05; **: *p* < 0.01; ***: *p* < 0.001; †: significant differences for caffeine versus placebo at the group level; #: significant differences detected between elite and recreational athletes at the supplementation level

A significant effect of the factor group was noted when depression measures were analyzed (F_1,16_ = 5.24; *p* = 0.036; ${\eta}_p^2$ = 0.25). Elite athletes awarded more points to the dimension depression (3.25 ± 0.93) than the trained-recreational athletes (1.30 ± 2.7). No significant supplementation by group interaction was observed (F_1,16_ = 1.474; *p* = 0.242; ${\eta}_p^2$ = 0.08) nor a significant effect of supplementation (F_1,16_ = 0.018; *p* = 0.894; ${\eta}_p^2$ = 0.001).

For the dimension anger, there was no significant interaction between supplementation and group (F_1,16_ = 1.481; *p* = 0.241; ${\eta}_p^2$ = 0.08) or any significant effects of supplementation (F_1,16_ = 0.165; *p* = 0.690; ${\eta}_p^2$ = 0.01) or group (F_1,16_ = 1.967; *p* = 0.180; ${\eta}_p^2$ = 0.11).

When measures of vigor were analyzed, a significant supplementation by group interaction was noted (F_1,16_ = 11.284; *p* = 0.004; ${\eta}_p^2$ = 0.41; Fig. 1b), accompanied by a significant effect of supplementation (F_1,16_ = 29.070; *p* < 0.001; ${\eta}_p^2$ = 0.64). However, no significant effect was detected of the factor group (F_1,16_ = 2.706; *p* = 0.119; ${\eta}_p^2$ = 0.14). Our post-hoc pairwise analysis revealed significantly greater vigor values for the elite than trained- recreational athletes after caffeine supplementation (16,5 ± 2.62 vs. 13 ± 3.02; *p* = 0.20; *d* = 1.24). Moreover, significantly higher vigor scores were obtained for the elite athletes when these were supplemented with caffeine rather than placebo (16.5 ± 2.62 vs. 12.63 ± 2.33; *p* < 0.001; *d* = 1.56). A significant effect of supplementation was also found on self-reported fatigue (F_1,16_ = 9.164; *p* = 0.008; ${\eta}_p^2$ = 0.36). Accordingly, participants showed higher fatigue levels after supplementation with placebo (4.89 ± 4.42) than CAFF (2.56 ± 2.77). There was no significant supplementation by group interaction (F_1,16_ = 3.299; *p* = 0.088; ${\eta}_p^2$ = 0.17) nor significant effect of group (F_1,16_ = 0.850; *p* = 0.370; ${\eta}_p^2$ = 0.05).

For confusion levels reported by the participants, there was no significant supplementation by group interaction (F_1,16_ = 1.516; *p* = 0.236; ${\eta}_p^2$ = 0.09), nor a significant effect of supplementation (F_1,16_ = 0.257; *p* = 0.619; ${\eta}_p^2$ = 0.02) or group (F_1,16_ = 1.035; *p* = 0.324; ${\eta}_p^2$ = 0.06).

As for the subjective vitality scale, a significant supplementation by group interaction was detected (F_1,16_ = 11.028; *p* = 0.004; ${\eta}_p^2$ = 0.41; Fig. 1c), along with a significant effect of supplementation (F_1,16_ = 22.863; *p* < 0.001; ${\eta}_p^2$ = 0.59) but no significant effect of group (F_1,16_ = 0.292; *p* = 0.597; ${\eta}_p^2$ = 0.02). Post-hoc pairwise comparisons revealed that elite athletes returned higher SVS scores after caffeine supplementation compared to placebo (40.63 ± 4.78 vs. 31.75 ± 7.78; *p* < 0.001; *d* = 1.41).

## Discussion

The present study was designed to compare the acute effects of CAFF supplementation on anaerobic performance, mood and perceived exertion in elite athletes and moderately-trained recreational athletes. Our findings indicate that the ergogenic effect of CAFF is determined by an athlete’s performance level but only in terms of variables related to mood state. Hence, elite athletes reported significantly higher tension levels following the intake of CAFF (+ 325%) than the intake of PLAC. Similarly, CAFF supplementation led to significantly higher measures of vigor compared to the effect of PLAC (+ 31%) but only in the elite athletes. Further, vigor levels reported after the intake of CAFF were significantly higher (+ 27%) in the elite athletes than recreational athletes. Finally, elite athletes also returned significantly higher SVS scores after the intake of CAFF compared to PLAC (+ 5.8%). In contrast, it emerged that the ergogenic effect of CAFF on measures of anaerobic performance were independent of the athletes’ training level.

In line with prior work, studies have shown that CAFF supplementation produces an increase in peak and average power and that this effect is not conditioned by the performance level of the athlete [19, 54, 55]. Consistently, we observed here that CAFF intake led to a shorter time needed to reach peak power in both experimental groups (− 9.9% in elite and 22.5% in trained-recreational athletes). These data suggest that an athlete’s training level does not modify the ergogenic effect exerted by CAFF supplementation on anaerobic performance (i.e., peak and average power). Our results are line with the ergogenic effect of CAFF observed both in elite athletes [13, 35, 36] and in recreational athletes [37], and also with the improved physical performance noted after the intake of CAFF in trained and untrained athletes [34]. In contrast, Collomp et al. reported an ergogenic CAFF effect in an anaerobic capacity field test (100 m freestyle swimming) in trained swimmers but not in untrained swimmers [33]. If we consider evidence suggesting that the amount of supplemented CAFF determines its potential ergogenic effect [56–58], it seems reasonable that this variable could explain the disparate results obtained in our study and the report by Collomp et al. [33]. While the dose of CAFF in the present study was personalized (6 mg/kg), Collomp et al. used a standard dose of 250 mg (~ 4.3 mg/kg) [33]. Moreover, as it has been established that CAFF supplementation produces a greater effect on movement velocity as the dose is increased and, especially, as the load is increased [59], the greater amount of CAFF used here could explain our different results to those of Collomp et al. [33].

Another explanation for the different results obtained by Collomp et al. could be the type of physical test used [33]. Thus, while we used a standard test to assess anaerobic capacity (Wingate test), Collomp et al. employed a test that was specific for the athletes tested (i.e., 100 m freestyle) [33]. This could explain why our trained-recreational athletes developed more peak power than the elite athletes, although other studies have detected no differences in physical performance between different-level athletes [60, 61] or between boxers and physically inactive individuals [62]. Notwithstanding, elite boxers were found to show improved anaerobic performance compared to amateur boxers in an arm cycle ergometry Wingate test [63, 64]. This could suggest that the higher peak power value recorded in our recreational athletes reflected the greater adaptation of these athletes to maximal efforts executed with the legs, regardless of the specificity of the anaerobic test performed according to the given sports modality. In future work, the ergogenic effect of CAFF should be examined in different level athletes (elite vs. trained-recreational) who practice the same sports activity in physical performance tests specific to the physiological and biomechanical needs of that sport.

The effects of caffeine on anaerobic performance may be explained by both peripheral and central mechanisms. At the peripheral level, the effects of CAFF supplementation, such as enhanced neuromuscular recruitment [14, 15] and increased bioavailability of calcium in the myoplasm [16], could explain the positive effect on power production by the muscle and thus explain the ergogenic effects observed in the Wingate test. Centrally, CAFF is an adenosine antagonist, increasing neurotransmitter synthesis [11, 12] and nervous system stimulation [65]. How these effects impact RPE and physical performance are still being explored [66]. Studies have shown that CAFF doses of 3–6 mg/kg give rise to improved performance and reduced RPE in sets of resistance exercise with submaximal loads [67], and physical condition tests in basketball players [68]. In Wingate tests, improved ergometry performance in the arms accompanied by a decline in RPE has been observed [69], as well as improved performance in the legs both in moderately-trained subjects [70] and trained judokas [71, 72]. We could not confirm any beneficial effects of CAFF on RPE here. However, the improved W_avg_ levels observed associated with a similar RPE, suggest that CAFF supplementation exerts its positive effects by allowing a greater workload for a given RPE [73, 74], since it would be reasonable to expect lower RPE values at CAFF condition (compared to PLAC) if similar performance were registered for both experimental conditions [75]. Accordingly, CAFF seems to modify the relationship between workload and RPE, reducing RPE for a given load [76]. Therefore, some of the ergogenic effects of CAFF could take place via a diminished sensation of fatigue induced by exercise [77].

As adenosine increases pain and tiredness perception while it diminishes arousal [10], CAFF supplementation, through its effects on adenosine, could have a positive effect on a person’s mood state [25, 78]. Some authors propose that an increased mood state via augmented tension, reflects an optimal emotional state to approach a physical task [48]. This determines that small increases in tension levels before exercise could lead to improved performance [79]. Our results indicate that CAFF supplementation has an effect in high-performance athletes. This is consistent with studies in which improved performance was accompanied by increases in tension and vigor and a reduction in fatigue in elite judo athletes [71, 72, 80]. In an attempt to explain such enhanced tension levels only in elite athletes, Lane et al. [81] and Lane and Jarret [82] argued that elite athletes are accustomed to high levels of tension and vigor, and that this leads to adaptation to variations met in situations of high physical demands to optimize the state of approaching a task [26, 30]. This situation is uncommon in recreational athletes. In a study examining fatigue perception, Paton et al. measured sprints in trained cyclists who trained under the effects of caffeine or placebo [83]. The authors noted that acute CAFF intake significantly reduced the feeling of fatigue when executing a repeated high-intensity exercise in elite cyclists, coinciding with the findings of studies conducted in moderately-trained [84] and recreational athletes [27]. In our study, the sensation of fatigue diminished significantly in both groups of athletes when taking caffeine as a supplement before conducting a maximum intensity task. This evidence of the effects of CAFF in increasing tension and vigor, and diminishing the sensation of fatigue in the case of elite boxers allows for adaptation to an optimal state for facing maximum intensity explosive actions.

In the depression dimension of the POMS questionnaire, the professional boxers scored higher than the recreational athletes. While several studies indicate that practicing sports improves mood in terms of improving depression [85, 86], two literature reviews [87]. reported that athletes of sports modalities possibly causing concussion (e.g., boxing), showed high prevalence of depression symptoms. Thus, repeated concussion episodes produced after a blow transmitting an inertial force to the brain can give rise to depressive symptoms [88]. This is attributable to a strong correlation observed between athletes with a history of concussion and abnormalities in frontal lobe alpha waves and symptoms of depression [89]. Studies have identified a substantial increase (~ 20%) in depression symptoms in athletes who have suffered concussion [90, 91]. Further, athletes of sports with a risk of concussion such as American football, rugby or boxing are 2 to 3 times more likely to suffer from depression than the general population [92–94]. This means it could be that the differences detected here between groups were linked to the sports modality of the high-performance athletes (i.e., boxing). Therefore, there is need to evaluate potential associations between concussion and depression symptoms in future studies, in which different athletes’ cohorts should be recruited. This would allow researchers to analyze potential underlying mechanisms for depression, in addition to broaden our knowledge regarding concussion-related mental disorders (i.e. anxiety), which have been previously studied in retired athletes [95, 96].

### Limitations of the study

Individual caffeine tolerance has been previously associated with decreased ergogenic effect of caffeine supplementation, resulting from an increased activity of the adenosine receptors, along with a decreased β-adrenergic activity [25]. Unfortunately, it was not possible for us to control for individual’s caffeine tolerance on this study, since participant’s average caffeine intake was not recorded. Therefore, habituation to caffeine may have been a potential confound variable on our study, and it should be controlled in future research aiming at comparing the ergogenic effect of caffeine between different populations.

Although a set of nutritional guidelines was given to each participant, in order to ensure similar proportions of ingested macronutrients, individual dietary compliance was not assessed. Thus, there is need for control of this variable in future research, in order to fully cancelled potential interactions between supplementation and nutritional factors.

Finally, recruitment criteria associated with strength parameters (i.e. bench press 1RM greater than body weight, and 1RM 1.5 times body weight in full squat) were only applied for the recreational group. Thus, it cannot be fully discarded that between-group differences found for peak and average power resulted from potential strength differences between experimental groups. It is recommended that future research controls for this potential confound variable.

## Conclusions

Supplementation with 6 mg/kg of CAFF had an ergogenic effect on anaerobic performance, improving average power, peak power and the time needed to reach peak power in elite and in trained-recreational athletes. These improvements took place without a concomitant increase in RPE. Further, CAFF supplementation led to considerable improvements in factors contributing to mood state such as tension, vigor and vitality perception, but only in the elite athletes. In order to be able to generalize our conclusions to different athletes’ populations, future research is needed aiming at comparing caffeine’s ergogenic effect on recreationally-trained athletes and elite athletes from different sport modalities.

## Data Availability

The datasets used and/or analyzed during the current study are available from the corresponding author on reasonable request.

## Abbreviations

ANOVA: analysis of variance
BMI: body mass index
CAFF: caffeine
PLAC: placebo
POMS: profile of mood states
RM: repetition maximum
RPE: rating of perceived exertion
RPE_cardio_: exertion perceived at the cardiorespiratory level
RPE_general_: exertion perceived at the general level
RPE_muscular_: exertion perceived at the level of the legs
SD: standard deviation
SVS: subjective vitality scale
Time W_peak_: time taken (s) to reach peak power
W: power
W_avg_: average power
W_min_: minimum power output
W_peak_: peak power
